# Ex Ante Assessment of Returns on Research Investments to Address the Impact of Fusarium Wilt Tropical Race 4 on Global Banana Production

**DOI:** 10.3389/fpls.2020.00844

**Published:** 2020-07-06

**Authors:** Charles Staver, Diemuth E. Pemsl, Lars Scheerer, Luis Perez Vicente, Miguel Dita

**Affiliations:** ^1^Bioversity International, Montpellier, France; ^2^Instituto de Investigaciones de Sanidad Vegetal, Habana, Cuba; ^3^Bioversity International, Santiago de Cali, Colombia

**Keywords:** Fusarium wilt, banana, research priorities, ex ante, impact assessment

## Abstract

The spread of Fusarium *oxysporum* f. sp. *cubense* tropical race 4 (Foc TR4), causal agent of Fusarium wilt of banana (FWB), has been projected to reach 17% of the global banana-growing area by 2040 equaling 36 million tons of production worth over US$10 billion. This potential loss has fueled (inter)national discussions about the best responses to protect production and small-scale growers’ livelihoods. As part of a multi-crop ex ante assessment of returns on research investments conducted by the CGIAR Research Program on Roots, Tubers, and Bananas (RTB) from 2012 to 2016, four FWB research options were assessed: (i) improved exclusion, surveillance, eradication, and containment (ESEC) measures to reduce Foc TR4 spread, (ii) integrated crop and disease management (ICDM) to facilitate production of partially FWB resistant cultivars on Foc-infested soils, (iii) conventional breeding of FWB-resistant cultivars (CBRC), and (iv) genetically modified (GM) FWB-resistant cultivars (GMRC). Building on a risk index (Foc scale) predicting the initial occurrence and internal spread of Foc TR4 in 29 countries, an economic surplus (ES) model, cost-benefit analysis, and poverty impact simulations were used to assess impact under two adoption scenarios. All options yield positive net present values (NPVs) and internal rates of return (IRRs) above the standard 10% rate. For the conservative scenario with 50% reduced adoption, IRRs were still 30% for ICDM, 20% for CBRC, and 28% for GMRC. ESEC has IRRs between 11 and 14%, due to higher costs of capacity strengthening, on-going surveillance, farmer awareness campaigns, and implementation of farm biosecurity practices, which could be effective for other diseases and benefit multiple crops. The research investments would reach between 2.7 million (GMRC) and 14 million (ESEC) small-scale beneficiaries across Asia/Pacific, Sub-Saharan Africa, and Latin America/Caribbean. The options varied in their potential to reduce poverty, with the largest poverty reduction resulting from CBRC with 850,000 and ESEC with 807,000 persons lifted out of poverty (higher adoption scenario). In the discussion, we address the data needs for more fine-grained calculations to better guide research investment decisions. Our results show the potential of public investments in concerted research addressing the spread of Foc TR4 to yield high returns and substantially slow down disease spread.

## Introduction

The threat of the tropical race 4 of Fusarium *oxysporum* f. sp. *cubense* (Foc TR4), causal agent of Fusarium wilt of banana (FWB), to world banana supplies has been raised frequently in the popular press in the past several years^1^. These articles highlight Cavendish, the dominant banana cultivar group, which accounts for around 90% of current export production and is highly susceptible to Foc TR4 (Ploetz, 2005). However, other cultivars consumed and traded locally are also susceptible, although characterization is still ongoing (Hermanto et al., 2011; Zuo et al., 2018; Chen et al., 2019). Currently, Foc TR4 is present in 27 countries where thousands of hectares have been affected. A recent projection concluded that 17% of today’s banana growing area with an annual production of 36 million tons worth approximately US$10 billion at current prices could be lost over the next 20 years (Scheerer et al., 2018a). When the race 1 of this pathogen (Foc R1) threatened the export banana business during the period 1900–1950 (Stover, 1990), commercial producers successfully switched from the highly susceptible cultivar Gros Michel to the resistant Cavendish. Today, Cavendish constitutes about 50% of global banana production (FRuiTRoP, 2016) and the boxed banana postharvest supply chain is based completely on Cavendish requirements. While FWB ceased to be a concern for export banana growers, Foc R1 and R2 strains continue to spread and threaten banana-based livelihoods especially of smallholder farmers growing diverse susceptible cultivars. In response, contract growers of the highly FWB-susceptible Maça (Silk, AAB) cultivar in Brazil move production to clean soils every one to two crop cycles. Due to FWB in East Africa, small farmers have replaced Pisang Awak (ABB) used for banana juice with other cultivars. Gros Michel is still a preferred national market cultivar in Central America as an intercrop in shaded coffee, but an increasing number of producers have lost this income option because their fields are highly infected with the FWB pathogen and no efficient management options are available (Siles et al., 2013).

Frequent calls have been made for increased global investment to reduce the impact of FWB (Kema and Weise, 2013) and the Food and Agricultural Organization of the United Nations recently launched a global initiative which is seeking donors to invest US$98 million for a concerted response (FAO, 2017). However, while there is certain consensus that the threat of Foc TR4 is real and needs to be addressed, agreeing on a global investment strategy is challenging. Often the collapse of banana production is presented as imminent through words like banana extinction, apocalypse, and the end of banana (see news headlines in Footnote 1). However, the example of Foc R1 suggests that decades may pass until disease spread impacts large numbers of smallholder producers thereby critically affecting supplies. Cook et al. (2015) project high losses, even with a slow rate of spread, for bananas in Australia due to Foc TR4, where production is primarily based on Cavendish. Clearly, the threat is huge to the Cavendish-based production sector both for export and large internal markets like China, India, Indonesia, Brazil, and Mexico, because Cavendish has been shown to be highly susceptible to Foc TR4 and production is often concentrated in large monocultures in coastal lowlands subject to flooding, an accelerator of the pathogen spread (Dita et al., 2018).

The spread of the disease across China with the loss of over 100,000 hectares of production area and into four other countries of the Mekong sub-region over two to three decades as contract Cavendish growers have sought out clean lands for production illustrates a worst case scenario in the absence of action (Zheng et al., 2018). However, the extent of the threat to smallholder systems with a wide diversity of cultivars and cropping systems is uncertain. Is this sector protected by its diversity? Is the pathogen spread slower than in highly intensive monocrops, but inexorable, even for cultivars which show partial tolerance?

Breeding has been proposed as the most viable response through genetic modification (Loeillet, 2019) and with even greater promise in gene editing (Dale et al., 2017). Somaclonal variants tolerant to Foc TR4, a strategy pioneered in Taiwan (Hwang and Ko, 2004) and field tested in more tropical regions (Molina et al., 2016), have been taken up commercially and several national programs have established on-going selection. However, the touted success of these somaclonal Cavendish in infested areas (Molina, 2016) and the promise of short-term success of gene-edited cultivars cited above may put at risk the investment on exclusion and containment of FWB. Other authors (e.g., Dita et al., 2018) emphasize the importance of research on surveillance, exclusion, and containment to slow down and limit the spread as well as research on cropping systems management to both facilitate the production of susceptible cultivars and increase the durability of new resistant clones in infested lands.

Globally, the public research budget to address opportunities and threats to agricultural production has increased faster in larger countries, while smaller countries have faced many competing expenses (Beintema and Elliott, 2011). At the same time, the agricultural research agenda now addresses an expanded list of topics beyond increasing or maintaining productivity, such as climate change, environmental conservation, and poverty reduction (Place et al., 2013). In addition to conducting ex post impact assessment studies to demonstrate to donors and the global public that invested funds have generated (large) positive returns (e.g., Renkow and Byerlee, 2010), ex ante assessments (with different levels of rigor and formality) that determine *a priori* expected returns on investment have been widely applied. These studies are generally used to support and justify strategic research portfolio decisions in order to maximize the benefit of limited resources (see, e.g., case studies in Raitzer and Norton, 2009) and respond to increased up-front accountability demands from donors (Pardey et al., 2016). Quantitative ex ante studies have been conducted for a range of different agricultural technologies and locations (recent examples are Ainembabazi et al., 2015; Komarek et al., 2019).

As a global research partnership, CGIAR is the world’s largest international agricultural research network, implementing 15 thematic Consortium Research Programs (CRPs) in collaboration with 1000+ partners worldwide^2^. The Research Program focusing on Roots, Tubers, and Bananas (RTB) comprises five research centers (Bioversity International, the International Center for Tropical Agriculture (CIAT), the International Institute of Tropical Agriculture (IITA), and the French Agricultural Research Center for International Development (CIRAD) who work with more than 360 other partners. RTB, as a new program in 2012, was requested to conduct a priority assessment and implemented a rigorous, harmonized quantitative ex ante study for its five major crops (banana, cassava, potato, sweet potato, and yam).

The RTB centers and institutes working on banana (Bioversity, IITA, and CIRAD) together with national banana programs articulated through four regional banana networks carried out a banana priority assessment as part of the multi-crop cross-Center effort. The assessment started with a participatory elicitation of major constraints and opportunities to (small-scale) banana production, processing, and marketing. The results of this global expert consultation with responses from 523 banana experts are summarized in an RTB publication (Pemsl et al., 2014). Globally, respondents ranked FWB fourth in this survey behind pest and disease-infected planting material, black leaf streak, and water deficits. In a subsequent workshop held in April 2013, 34 banana scientists, representing different geographic regions and areas of expertise, proposed initial research lines to address these major yield constraints. Ex ante analysis for eight of these research lines, five for banana breeding and three for crop management, was completed as part of the RTB priority assessment (Pemsl and Staver, 2014). Subsequently, four research options addressing the threat of FWB were assessed. Since the time of the assessment, Foc TR4 has spread to more countries in South and South-East Asia and Africa as well as Latin America^3^, increasing the urgency to invest in FWB research guided by a systematic and quantitative priority assessment.

We have three objectives in this paper:

1) Describe the methodological approach, data compilation, and results of the ex ante assessment of FWB research lines (incl. returns on investment and poverty impacts);
2) Assess the validity of the results with reference to their use by policy makers and funders and identify priority areas for future data collection and curation and complementary research for follow-up studies;
3) Discuss steps to improve the use of priority assessment studies to guide research funding decisions on FWB.

## Materials and Methods

The analytical framework used for the quantitative ex ante assessment follows the methodology used in the wider RTB priority assessment study across crops and is described in Alene et al. (2018). For the assessment of the Foc research options, these steps comprised the selection and detailed description of research options, compilation of data and parameter estimation, the quantification of potential impacts using a partial equilibrium economic surplus (ES) model and subsequent cost-benefit analysis, sensitivity analysis, and an online stakeholder feedback survey to validate parameter assumptions.

### Selection and Description of Research Options

In order to narrow down and describe the specific research options to be assessed, we clustered research interventions addressing Fusarium wilt around three general themes: (i) preventing the spread of the disease (especially Foc TR4) to currently unaffected regions/countries through research on (and implementation of) improved exclusion, surveillance, eradication, and containment (ESEC) measures; (ii) research on integrated crop and disease management (ICDM) to recover banana yields in areas affected by (all strains of) Fusarium; and (iii) research focused on developing banana cultivars resistant to Fusarium wilt. There are two fundamentally different approaches to developing resistant varieties: conventional breeding using the genetic diversity of banana or genetic modification of susceptible cultivars of economic importance, with the latter likely being applicable only for a smaller area due to country biosafety regulations.

Based on these considerations, four distinct potential research options to address FWB were selected and quantitatively assessed for this study:

– Improved ESEC measures to avoid Foc TR4 spread (ESEC);
– Integrated crop and disease management to reduce impact of Foc TR4 (ICDM);
– Conventional breeding for FWB-resistant banana cultivars (CBRC);
– Genetically modified (GM) FWB-resistant banana cultivars (GMRC).

The adoptable, public good innovations resulting from the research in the form of knowledge, practices, and technologies were formulated for each research option and the specific research agenda was detailed (Table 1). This provided a scope of work for each research option, required to budget expected research costs for the cost-benefit analysis. Even though some topics, e.g., epidemiology, pathogenicity, diagnostic protocols, clean seed, and mapping relate to more than one research option (see ESEC and ICDM overlap in Table 1), we costed each option separately so they can later be compared. If investment in both options occurred, there would be substantial synergies that would result in lower research costs.

**TABLE 1:** Description of the four assessed research options to address Fusarium wilt of bananas.

**Research option**	**Improved exclusion, surveillance, eradication and containment (ESEC)**	**Integrated crop and disease management (ICDM)**	**Conventional breeding of Fusarium resistant banana cultivars (CBRC)**	**Genetically modified Fusarium resistant banana cultivars (GMRC)**
**Adoptable innovation**	(Improved) exclusion, surveillance, containment, and early eradication measures on farm, community, national, and international level	Crop and disease management package	High yielding and market accepted Fusarium resistant varieties	High yielding and market-accepted genetically modified (GM) Fusarium resistant varieties

**Research agenda**	• Strengthen science-based risk analysis protocol for Foc movement for local, national, regional, and intercontinental use • Develop/improve protocol to produce Foc-free planting material from tissue culture (TC), suckers, and macro propagation • Develop model for Foc epidemiology and pathogenicity and more efficient tools for epidemiological studies • Determine pathogen population structure, cultivar-specific disease intensity, and current distribution of Foc populations in key banana producing countries • Develop and optimize diagnostic protocols for TR4 and other relevant Foc strains Evaluate susceptibility/resistance of major cultivars to Foc TR 4 and other races		• Prospection for new sources of resistance to Foc in germplasm collection, including breeding lines • Identify and characterize resistance genes (and molecular markers) to support breeding processes including Marker Assisted Selection • Generate diploid pre-breeding lines with Foc resistance (emphasis on TR4) for major cultivar groups • Develop efficient protocols for phenotyping of breeding lines	• Identify pathogenicity factorsand defense/resistance genes and develop cisgenic and/or trans-genic constructs to generate Foc resistant bananas cultivars • Develop GM banana cultivars with Foc resistance • Phenotype GM bananas lines for Foc resistance at greenhouse level • Evaluate and select commercial GM lines resistant to Foc on multi-site field experiments
			
	• Validate efficient surveillance protocols to detect, delimitate, and monitor Foc spreading • Understand risk and pathways of Foc dissemination in soil, suckers, humans, other banana parts, diverse agricultural and non-agricultural practices within country, across borders, and between continents • Determine effectiveness of different eradication and isolation procedures for first detected Foc affected banana plants in Foc-free areas	• Identify and evaluate cover crops, intercrops, and other agronomic and soil management practices that suppress or accelerate Foc in banana and clarify mechanisms involved • Understand functional diversity of suppressive vs. conducive soils in banana production contrasting biological, physical, and chemical properties • Screen and characterize root-associated microorganisms w/Foc suppressive and growth promotion capacity • Prototype integrated Foc management strategies based on biological inputs (incl. microorganisms), crop (incl. resistant genotypes, chemical fertilizers fine tuning), and cropping systems	• Employ conventionally breeding methods to develop bananas with Foc resistance • Strengthen protocols and develop somaclonal and clonal selection for Foc resistance in susceptible (and partially resistant) cultivars • Identify possible Foc resistant substitutes for the major susceptible market and food security cultivars and select for clones with superior traits • Evaluate and select resistant genotypes on multi-site field experiments • Evaluate and develop post-harvest and market oriented strategies	• Evaluate and develop post-harvest and market oriented strategies

Each research option has a distinct target domain, since each option focuses on or is applicable to certain cultivar groups and is thus (more) relevant in certain countries. We considered major banana producing countries in Asia and the Pacific, Africa, and Latin America and the Caribbean (LAC). Our focus was on countries with predominantly small-holder producers and a substantial dependency on bananas for livelihoods.

The research on ESEC is applicable to all six cultivar groups in 29 major banana producing countries threatened by Foc TR4 (Table 2). The agenda will contribute to the effectiveness of national plant protection offices, starting with a better understanding and assessment of risks of Foc TR4 introduction and spread (Dita et al., 2013; Biosecurity of Queensland, 2016). Field studies on movement of planting material, banana products, and soil and other practical experience (Pegg et al., 2019) will contribute to ESEC strategies. Basic information about the disease in the plant is central not only to ESEC, but to ICDM (Warman and Aitken, 2018) and should be expanded. Such basic knowledge is also applicable to a spatial model for the analysis of scenarios for different locations of first outbreak and the speed and likelihood of spread depending on the actions taken (see Flores et al., 2017, for an example on citrus greening disease). Such models will also contribute to more effective surveillance routines and routes and to more strategic actions for eradication and containment. Research also includes development of more effective measures of biosecurity both in large plantations and in zones of diversified small farms with bananas (Pérez Vicente, 2015; Kukulies and Veivers, 2017).

**TABLE 2:** Target domains for the assessed research options to address Fusarium wilt of bananas.

**Research option**	**Improved exclusion, surveillance, eradication, and containment (ESEC)**	**Integrated crop and disease management (ICDM)**	**Conventional breeding of Fusarium resistant banana cultivars (CBRC)**	**Genetically modified Fusarium resistant banana cultivars (GMRC)**
Target domain	Production areas of all six cultivar groups in countries in Africa, LAC, and Asia/Pacific where Fusarium Foc TR4 is either already present or will very likely spread in the near future	Production areas of all six cultivar groups in countries in Africa, LAC, and Asia/Pacific where soils are infested with Fusarium R1 and/or TR4	Production area of all six cultivar groups in Africa, LAC, and Asia/Pacific	Production area of “Cavendish AAA” in countries where local markets are important (export-oriented countries are less likely to adopt GM varieties due to political and consumer concerns in importing countries)

Applicable cultivars	Cavendish AAA; other AAA + Gros Michel + AA; East African Highland AAA; AAB Plantain; other AAB; ABB	***Foc TR4:*** •Cavendish AAA; other AAA + Gros Michel + AA; East African Highland AAA; AAB Plantain; other AAB; ABB (in Asia/Pacific and LAC)	• Cavendish AAA; other AAA + Gros Michel + AA; East African Highland AAA; AAB Plantain; other AAB; ABB	Cavendish AAA
		Cavendish AAA (in Africa)		
		***Foc R1:*** Other AAA and “other AAB” (in LAC and Asia/Pacific)		

Countries included in assessment	***Africa***: Burundi, Cameroon, Congo, D.R., Côte d’Ivoire, Ghana, Kenya, Mozambique, Nigeria, Rwanda, Tanzania, Uganda ***Asia/Pacific:*** China, India, Indonesia, Malaysia, Myanmar, Pakistan, Papua New Guinea, Philippines, Thailand, Vietnam ***LAC***: Brazil, Colombia, Costa Rica, Ecuador, Guatemala, Mexico, Nicaragua, Peru	***Africa***: Cameroon, Côte d’Ivoire, Ghana ***Asia/Pacific:*** China, India, Indonesia, Malaysia, Myanmar, Pakistan, Philippines, Thailand, Vietnam ***LAC:*** Brazil, Colombia, Costa Rica, Ecuador, Guatemala, Mexico, Nicaragua, Peru	***Africa:*** Burundi, Cameroon, Congo, D.R., Côte d’Ivoire, Ghana, Kenya, Mozambique, Nigeria, Rwanda, Tanzania, Uganda ***Asia/Pacific:*** China, India, Indonesia, Malaysia, Myanmar, Pakistan, Philippines, Thailand, Vietnam ***LAC:*** Brazil, Colombia, Costa Rica, Ecuador, Guatemala, Mexico, Nicaragua, Peru	***Africa:*** Burundi, Congo, D.R., Kenya, Mozambique, Nigeria, Rwanda, Tanzania, Uganda ***Asia/Pacific:*** China, India, Indonesia, Malaysia, Myanmar, Pakistan, Thailand, Vietnam ***LAC:*** Brazil, Mexico, Peru

The research on ICDM is primarily applicable to commercial production for national and export markets (Table 2). The deployment of cover crops, microbial organisms, systematic crop rotation, and careful rogueing was considered unlikely among small growers who grow bananas for home consumption and local sale. Delivery systems for inputs and seed are also often deficient where small growers predominate. Research to develop such ICDM approaches centers on healthy soils with capacity to suppress disease build-up and crop management to reduce crop susceptibility and to limit inoculum accumulation. Research results on specific bacterial antagonists of the pathogen (Pérez Vicente et al., 2009; Shen et al., 2015; Bubici et al., 2019), crop suppressive effects (Huang et al., 2012), and integrated systems approaches (Haddad et al., 2018; Huang et al., 2019) already provide evidence for the potential of this strategy. Basic knowledge on Foc populations, cultivar susceptibility, and more advanced quantitative diagnostic tools should underlie the applied management approaches. While the ICDM approaches are applicable for Foc races 1 and SR4, the calculation of returns only takes into account the recovery of losses due to Foc TR4 projected based on the risk index or FOC scale.

The development of CBRC is proposed to have the widest applicability after ESEC with relatively easy uptake for home consumption, local and national markets and export, as long as eating and cooking quality and handling traits are acceptable (Table 2). A broad range of varieties for different cultivar groups is proposed. Different improvement approaches are considered (Table 1) based on improved varieties already available through conventional breeding by Embrapa, the Brazilian Agricultural Research Corporation (Silva et al., 2013), clonal selection in Cavendish by the Taiwan Banana Research Institute already cited, and mutation breeding in Australia (Smith et al., 2006). Screening for resistance among existing lines continues to cover more and more *Musa* diversity (Zuo et al., 2018; Chen et al., 2019; García-Bastidas et al., 2019). Important areas of work include screening for sources of resistance, more effective phenotyping tools, development of pre-breeding lines, marker-assisted crossing, and multi-site evaluation trials.

The development of GMRC is projected only for Cavendish with application to countries with limited export, but relatively large national markets. Current status of biosafety regulations and laws on GM crops were not taken into account but could reduce the number of countries included. The use of resistance genes for cis and trans modification and the identification of genes linked to resistance to guide gene editing have already advanced beyond proof of concept (Dale et al., 2017; Maxmen, 2019). Resulting materials are screened for resistance to all Foc races in greenhouse and then field trials with evaluation of postharvest handling and taste qualities are conducted.

### Data Compilation and Parameter Estimation

Data required for our ex ante assessment were collected from three general sources: statistical databases, other published sources, and expert estimates. For consistency, we followed the same procedure for compiling and cleaning/adjusting data that was used in the previous assessment of RTB banana research options that is described in detail in Pemsl and Staver (2014). To facilitate the disaggregation of production data by cultivar group, we used information from FRuiTRoP (2012) in addition to the FAO^4^ statistics. To avoid bias due to annual abnormalities, we computed a 3-year average for banana yield and price for each country included (using 2010–2012 data for consistency with the previous assessments, older data if necessary). Since FAO does not separate production from large scale, commercial plantations from (semi−) subsistence production under smallholder conditions, some yield figures for cultivar groups other than Cavendish for countries with sizable banana export industry were capped using expert judgment to reflect smallholder conditions. Yield and production data were then used to compute the banana production area for each included country (see Table 3).

**TABLE 3:** Estimated banana production area lost due to Fusarium wilt over time (by country and region).

**Country**	Banana production area*[‘000 ha]	**Banana production area lost due to Fusarium wilt [% of total]over time assuming 50% internal spread rate once in country (loss with 25% spread rate in parentheses if applicable)**					**Banana production area lost [‘000 ha] in 25 years due to FW with 50% internal spread (25% spread)**
		**2019 (year 5)**	**2024 (year 10)**	**2029 (year 15)**	**2034 (year 20)**	**2039 (year 25)**	
**Africa**							
Burundi	371.05	0	3	7 (6)	12 (10)	20 (15)	75.1 (45.5)
Cameroon	184.41	0	3	7 (6)	12 (10)	20 (15)	37.6 (27.3)
Congo, D.R.	391.62	0	0	4	11 (9)	19 (15)	74.4 (60.5)
Côte d’Ivoire	411.19	0	2	5 (5)	10 (8)	17 (12)	68.0 (49.1)
Ghana	191.75	0	0	4	9 (8)	16 (13)	30.2 (24.5)
Kenya	80.49	0	1	3 (3)	7 (5)	11 (8)	8.8 (6.3)
Mozambique	27.86	6	14 (12)	25 (20)	39 (29)	55 (38)	15.3 (10.7)
Nigeria	455.55	0	0	1	1 (1)	3 (2)	12.6 (10.1)
Rwanda	343.64	0	0	1	3 (3)	6 (5)	19.7 (15.8)
Tanzania	537.68	0	4	10 (9)	18 (15)	29 (21)	156.7 (115.6)
Uganda	1866.25	0	0	1	2 (1)	3 (2)	55.1 (44.2)
**Subtotal**	**4861.49**						**553.5 (418.7)**
**Asia/Pacific**							
China	398.19	8	19 (17)	34 (28)	52 (39)	71 (51)	283.4 (202.3)
India	1858.28	0	0	2	5 (4)	9 (7)	163.3 (131.8)
Indonesia	320.03	4	10 (9)	18 (14)	29 (21)	43 (29)	137.6 (91.6)
Malaysia	56.82	2	5 (4)	9 (7)	15 (11)	23 (15)	13.2 (8.5)
Myanmar	65.43	0	8	18 (17)	33 (27)	50 (38)	32.8 (24.7)
Pakistan	31.98	8	19 (17)	33 (27)	51 (39)	71 (50)	22.6 (16.1)
Papua New Guinea	45.18	0	4	10 (9)	18 (14)	29 (21)	13.1 (9.6)
Philippines	391.88	8	19 (17)	34 (28)	52 (39)	71 (51)	278.9 (199.1)
Thailand	132.08	0	8	19 (17)	33 (27)	50 (38)	66.6 (50.2)
Vietnam	102.17	8	19 (17)	34 (28)	52 (39)	71 (51)	72.7 (51.9)
**Subtotal**	**3402.04**						**1084.1 (785.7)**
**Latin America and Caribbean (LAC)**							
Brazil	498.45	0	0	0	1	2 (2)	12.0 (10.8)
Colombia	461.43	0	0	1	2 (1)	3 (2)	13.8 (11.1)
Costa Rica	61.22	0	0	1	2 (2)	4 (3)	2.7 (2.1)
Ecuador	266.88	0	0	1	2 (2)	4 (3)	10.2 (8.2)
Guatemala	50.55	0	0	0	2	8 (4)	4.1 (2.0)
Mexico	86.31	0	0	0	1	2 (2)	2.0 (1.7)
Nicaragua	14.46	0	0	0	0	1	0.1 (0.1)
Peru	120.83	0	0	0	1	2 (2)	2.5 (2.1)
**Subtotal**	**1560.13**						**47.3 (38.1)**
**ALL**	**9823.66**	0.9	2.8 (2.6)	6.3 (5.5)	11.1 (8.8)	17.1 (12.6)	**1684 (1242)**

Source: *Source for production area FAO STA (averages of 2010–2012 values) and FRuiTRoP (2012) Results of the “Foc Scale” as part of the Strategic Assessment of Banana Research Priorities (Scheerer et al., 2018a, b).

To populate the poverty impact model, we relied on data included in the World Development Indicators^5^, namely, the most recent information for each included country on poverty prevalence (total population and poverty rate) and (agricultural) gross domestic product (GDP) by country.

### Predicting Pathogen Spread

The benefit of the FWB research interventions is the yield loss avoided^6^ by either preventing or delaying the spread of the disease (ESEC) to an area, (partially) recovering yields in areas with infested soils through crop or disease management (ICDM), or replacing susceptible with resistant banana cultivars that are not affected by the disease (CBRC and GMRC). The magnitude of the benefits from each of these options largely depends on the scale and pace of disease spread, i.e., the size of the affected area that constitutes the target domain for the intervention.

In the absence of an established epidemiological model to predict the future spread of Foc TR4, we relied on the risk-index model developed by Scheerer et al. (2018a) to project the future disease spread and thus determine the expected yield losses that could be avoided by investing in the four research options. The risk-index model consists of two steps where for each country a score is assigned based on (i) the likelihood of initial outbreak of Foc TR4 (time lag in years) and (ii) the internal spread rate of the disease (disaggregated per cultivar type) once present in the area. Factors considered when scoring for the time lag for Foc TR4 to reach a country include the importance of mono-cropped Cavendish, global banana traffic to and from a country, quality of borders and internal plant quarantine measures, and land and other links to countries where Foc TR4 is already present. The higher the aggregated score for a country, the shorter the time lag for Foc TR4 to be introduced and established in the country. The internal spread rate is scored based on three factors: the quality of internal quarantine measures, the importance of Cavendish, and the importance of banana for research investment and public policy. The higher the aggregated score for a country, the faster the spread and thus the higher the expected loss of banana production due to Foc TR4 with differentiated losses depending on cultivar types. Loss of production was proposed between 1 and 8% of area planted during the first 5 years after detection depending on cultivar group and the country score for internal spread risk. Assuming an accelerating expansion of the disease-affected area, especially in the early years, in each successive 5-year period after first detection, the spread rate was calculated to increase by 50%. In a second, more conservative scenario, the spread rate only increased by 25% for each successive 5-year interval.

In Table 3, we show the projected banana area lost to FWB using the risk index for each country over time (2014–2039, in 5-year intervals) in percent of the national banana production area lost. The last column gives the absolute area rendered unsuitable for production due to FWB in the absence of interventions thus constituting the target domain for our research interventions. The projected national losses are organized by region to illustrate the projected impact of the disease in different parts of the world over the next 25 years. The large effect in Asia/Pacific is due to the fact that Foc TR4 has already been detected in several countries in this region and internal spread has progressed. Though the current spread of the disease is more limited in Africa, the results indicate the potential for very high negative impact especially on smallholders. In the model results, spread onset was most delayed for LAC. However, the recent detection of Foc TR4 in Colombia is expected to accelerate the spread in LAC and shows how difficult the prediction of initial outbreak is.

**FIGURE 1:**
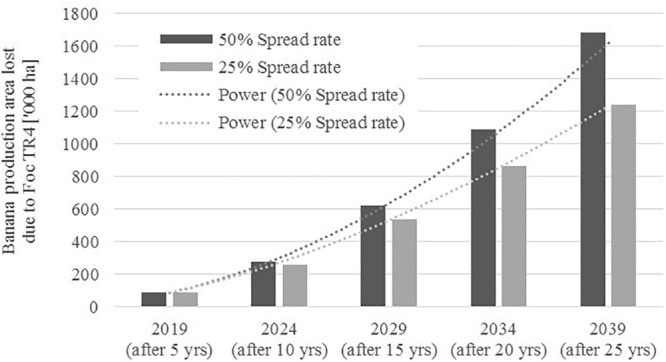
Projected loss of global banana production area due to FW over time (two scenarios). Source: Adapted from Scheerer et al. (2018a).

The accelerating nature of the spread over time as well as the slowing effect of a more conservative internal spread assumption is visualized in Figure 1.

### Quantifying the Potential Impact of Research Options

Following the methodology used in the multi-crop RTB priority assessment, we simulated adoption of the innovations resulting from ICDM, CBRC, and GMRC over time based on the target domain and estimated parameters on adoption lag (time until first adoption of innovation), the adoption pace, and ceiling. The adoption pace is indicated by the time until full adoption. The adoption ceiling is defined as the maximum share of the total production area affected by the disease on which the innovation(s) will be adopted. The definition of adoption is different for the ESEC research option, since the decision to implement is taken on the national (or even regional) level instead of the individual producer level and thus either none or all of the (affected) production area in the country benefits. Table 4 gives an overview of the parameter assumptions used in the assessment.

**TABLE 4:** Summary of parameter estimates and assumptions.

**Parameter**	**Improved exclusion, surveillance, eradication, and containment (ESEC)**	**Integrated crop and disease management (ICDM)**	**Conventional breeding of FWB resistant banana cultivars (CBRC)**	**Genetically modified FWB resistant banana cultivars (GMRC)**
Change in yield (%) = avoided yield loss	100	80	100	100
Production cost change (%)	1	20	NA	NA
R&D costs (US$ million)^1^	16.2	30.5	47.7	8.5
Dissemination costs (US$ha of new adoption)	50	80	50	50
Additional costs	***Establishing quarantine system***^2^ : US$50/ha ***Maintaining quarantine system***: US$5/ha/year prior to Foc arrival and US$10/ha/year with Foc	NA	NA	NA
Adoption ceiling (% of target domain)	100^3^	30–50	80	40
Adoption ceiling (% of total national production area)	2–51	0.3–25	0.8–41	0.1–18
Research lag (years)	8	10	17	12
Time between first adoption and adoption ceiling (years)	10	15	15	15
Chance of research success (%)	80	90	60	40
Chance of national uptake^4^ (%)	80	25, 50, or 75^5^	90	70

**^1^Matched 1:1 with additional costs expected to occur at the national level for the cost-benefit analysis. ^2^In year 5 for countries with high importance of banana and in year 10 for countries with low importance of banana. ^3^Given that quarantine and surveillance measures are executed at the national level, we assumed that all farmers “adopt” or benefit from the technology once the country implements the quarantine scheme, i.e., “adoption” is 100% of the target domain. ^4^Probability of successful up-take of the technology by national level (extension) agencies to enable adoption. ^5^Depending on the importance of banana to national public policy and national research and extension investments.**

The largest adoption area reached over the assessment period represents the reach of the intervention and is reported as one impact indicator in the results section. This largest adoption area figure is also the basis for the computation of the number of beneficiaries, an additional impact indicator. The conversion is conducted by division with country specific estimates of the average banana area per household and multiplication with the country specific average household size (RTB, 2011).

To quantify the benefits from adopting the innovations developed under each of the research options at the national level, we used a partial equilibrium ES model estimated over a 25-year period (2014–2039). This quantitative approach of computing the ES resulting from a research-induced supply shift is a standard procedure in the agricultural economics and impact assessment field (see, e.g., Alston et al., 1995) and has been used in many previous studies (e.g., Alene et al., 2009, 2018; Fuglie and Thiele, 2009). We assumed elasticities of supply and demand to be 1 and 0.5, respectively, across all technologies and countries due to lack of other information.

In the subsequent cost-benefit analysis, we discount the benefits computed in the ES model (i.e., apply an interest rate to account for the difference in time when benefits occur) and compare them with the discounted costs associated with each research options. The estimated research and development (R&D) costs for each research option (Table 4) were doubled for the cost-benefit analysis to account for (in-kind) contributions of national partners. Additionally, dissemination costs based on the marginal annual adoption area were included. Dissemination costs were set at US$50 per hectare for improved varieties and US$80 per hectare for knowledge intensive innovations such as crop or pest management practices. For ESEC, additional costs were included for (i) establishing the quarantine system in the amount of US$50 per hectare reflecting the initial capacity strengthening effort and (ii) maintaining the quarantine and surveillance system in the amount of US$5 per hectare and year prior to Foc TR4 arrival and US$10 per hectare and year once Foc TR4 is present.

The key indicators resulting from the cost-benefit analysis are the net present value (NPV) and the internal rate of return (IRR) on the investment.

In addition to computing the standard economic indicators NPV and IRR, we also assessed the potential impact of each of the research options to reduce poverty. To do so, we estimated the marginal impact of an increase in the value of agricultural production on poverty reduction using elasticities of agricultural productivity growth. We applied the regional elasticities proposed by Thirtle et al. (2003) who found that a 1% growth in agricultural productivity reduces the number of rural poor by 0.72% in Africa, 0.48% in Asia, and 0.15% in LAC. Following Alene et al. (2009), we calculated the reduction in the number of poor by considering the estimated economic benefit of the respective research option as an increase in the agricultural production value.

### Sensitivity Analysis of Results

Ex ante assessments by their nature predict uncertain future outcomes of potential investments and results are based on (expert) estimates of the costs and effects. Results have notoriously been too optimistic with regard to the benefits while underestimating (unknown) costs and problems. Alston et al. (1995) proposed sensitivity analysis to remedy this situation. In order to probe the robustness of results and take into account the tendency of technical experts to be overly optimistic with regard to the performance of and demand for their technologies, we embedded different adoption scenarios in the assessment. We also conducted sensitivity testing for additional key variables, focusing on parameters elicited from experts rather than model inherent parameters (such as elasticities and discount rate) or parameters populated based on statistics (e.g., banana production area, yield, or farm-gate prices). In order to keep the number of scenarios manageable, we focused on the three most crucial parameters which at the same time seem most prone to overly optimistic assumptions: (1) adoption area of the new technology, (2) time when adoption starts, and (3) magnitude of the farm-level benefit realized when adopting the technology.

For ICDM, CBRC, and GMRC, we assessed two different adoption scenarios: the first uses the adoption ceiling estimated by experts, while the second one is more conservative and uses a 50% reduced adoption assumption. Subsequently, we tested additional scenarios with delayed adoption and reduced effects for these three research options.

Since adoption is either 0 or 100% at the national level for ESEC, we constructed three increasingly cautious scenarios in order to test sensitivity of the results to changes in key parameters (see Scheerer et al., 2018b). For Scenario 1, we assume that ESEC will lead to a doubling of the arrival time (e.g., the disease will first occur in a country after 10 instead of 5 years) and a 50% reduced increase in spread rate once Foc reaches the country (i.e., 12.5 instead of 25% increase of spread rate every 5 years). Scenario 2 has a less delayed first arrival [Scenario 1 minus 5 years, i.e., 5 years earlier than under Scenario 1 in our example after (5 years × 2) – 5 = 5 years] and the same reduced increase in spread rate of 12.5%. Scenario 3 has the same less delayed arrival from the second scenario and a small reduction in the increase in spread rate (18.75 instead of 25%) once the disease breaks out in a country.

## Results

### Economic Impact

The assessment results show that all research options for FWB generate positive NPVs under all adoption scenarios (Table 5), indicating that investment in all research options is profitable. The magnitude of NPVs, however, varies considerably across options ranging from US$35 million for the most conservative scenario of improved quarantine and surveillance measures to reduce Foc TR4 spread (ESEC) to slightly over US$1 billion for ICDM to facilitate commercial production of partially Foc-resistant cultivars on Foc-infested soils (expert adoption assumption scenario). Since R&D costs, or the level of investment required, vary substantially across research options (US$8.5—47.7 million), the NPVs cannot be used to rank the research options. Instead, the IRRs (the interest rate realized on the invested amount) are a preferred measure for ranking alternative technologies.

**TABLE 5:** Results of ex ante assessment: adoption area, NPV and IRR, beneficiaries, and poverty reduction.

**Research option w/adoption scenario**	**Adoption area after 25 years (’000 ha)**	**All benefits**		**Number of beneficiaries (‘000 persons)**	**Poverty reduction (‘000 persons)**
		**NPV (US$ million)**	**IRR (%)**		
**ESEC—Improved exclusion, surveillance, eradication, and containment**					
Scenario 1*	404	260.84	14	9107	807
Scenario 2**	363	156.69	13	8237	714
Scenario 3***	300	35.10	11	6654	615
**ICDM—Integrated crop and disease management**					
*Expert estimated adoption scenario*	344	1040.29	36	7875	157
*50% reduced adoption scenario*	170	501.08	30	3926	79
**CBRC—Conventional breeding of FWB resistant banana cultivars**					
*Expert estimated adoption scenario*	593	418.54	25	14,040	850
*50% reduced adoption scenario*	297	183.36	20	7020	422
**GMRC—Genetically modified FWB resistant banana cultivars**					
*Expert estimated adoption scenario*	127	286.03	34	2743	89
*50% reduced adoption scenario*	63	137.02	28	1371	44

NPV calculated using a real interest rate of 10%. The adoption area of ESEC represents the area after 25 years where losses could be avoided due to the execution of the quarantine and surveillance measures at a national level. *Doubled arrival time and 50% reduced increase of spread rate (12.5%) once Foc reaches the country compared to counterfactual (no intervention); **Arrival time as in Scenario 1 minus 5 years; 50% reduced increase of spread rate (12.50%) once Foc reaches the country; ***Arrival time as in Scenario 1 minus 5 years; 25% reduced increase of spread rate (18.75%) once Foc reaches the country.

All assessed FWB research options yield positive IRRs that are above the standard 10% interest rate (Table 5). Even under the lower adoption scenario the IRRs are positive and mostly well above 10%. Again, there is considerable variation in the return on investment among research options and adoption scenarios. ESEC Scenario 3 yields an estimated 11% return on the investment while the higher adoption scenario for ICDM reaches an estimated 36% IRR. The three ESEC scenarios show the lowest returns on investment, just slightly above the 10% threshold. These lower returns result from additional cost variables we included compared to the other research options. In addition to the R&D costs (matched 1:1 with national partner costs for the assessment) and the dissemination costs included for all research options, we added the costs of establishing quarantine systems reflecting the initial capacity strengthening effort and the costs of maintaining the quarantine and surveillance system. The resulting costs during the first ten years are exceptionally high, thereby lowering the IRR. At the same time, we did not include any additional benefits resulting from reduced or prevented losses due to pests and diseases other than Foc, that would very likely result from strengthening national level of surveillance and quarantine systems. We consider our results for ESEC to be very conservative.

Table 5 displays the estimated area on which the new technologies will be adopted under each of the adoption scenarios. In the case of ESEC, since the adoption decision takes place on the national level, the adoption area represents the area after 25 years for which losses could be avoided (in this case all area affected) due to the execution of the quarantine and surveillance measures at a national level. In comparison, the adoption area for the other three research options is the area on which farmers apply the new technologies and thus individually avoid or reduce losses. The estimated adoption area translates into the number of people that benefit from the new technologies which is highlighted in the second last column of Table 5. These figures are based on the largest adoption area reached over the assessment period.

The estimates show that conventional breeding of FWB-resistant cultivars (CBRC) reaches the largest number of beneficiaries across all research options under both the higher (14 million beneficiaries) and lower adoption scenario (7 million beneficiaries) because of the largest estimated adoption area. The investment in breeding GMRC reaches the lowest number of beneficiaries (2.7 million and 1.4 million beneficiaries for the two adoption scenarios, respectively) due to the assumption that countries with export-oriented banana production would not adopt GM varieties due to political and consumer concerns in importing countries. Similar to the NPV and IRR results, these numbers should be interpreted in combination with the size of the investments required for each research option.

### Impact on Poverty

The last column of Table 5 shows the poverty reduction impact of the different research option, measured as the estimated number of persons lifted out of poverty. This indicator is largely driven by the total economic benefits, national poverty rates, and region-specific elasticities of poverty reduction with respect to agricultural productivity growth. With Africa having the highest poverty rates and largest poverty elasticity, the poverty reduction measure thus favors research options that generate a larger part of the benefits in Africa compared to the other regions. This partly explains why the estimated impacts on poverty reduction are highest for ESEC (615,000–807,000 people lifted out of poverty based on the scenario) and CBRC (850,000 and 422,000 for the two adoption scenarios, respectively), whereas the investment in GMRC has the lowest poverty reduction effect. The lower importance of Cavendish cultivars in Africa also contributes to these lower adoption figures. Research options that generate comparable global economic benefits may have different poverty reduction impacts depending on share of benefits generated in countries in Africa.

### Impact by Region

We estimated the regional distribution of the adoption area (Table 6) and find that most adoption occurs in Asia/Pacific (45–92%) followed by Africa (2–44%). The regional benefits are determined by the extent of spread over the period of the calculation with only small areas affected in Latin America^7^. The benefits are mainly determined by the adoption area, but also other parameters used in the model, such as cost effects, yield levels (which are currently much higher in LAC compared to most countries in Africa), crop prices, and likely success rate.

**TABLE 6:** Results—regional breakdown of adoption area.

**Technology**	**Adoption area after 25 years**						
	**Africa**		**LAC**		**Asia/Pacific**		**ALL**
	**(’000 ha)**	**Share (%)**	**(’000 ha)**	**Share (%)**	**(’000 ha)**	**Share (%)**	**(’000 ha)**
**ESEC—Improved exclusion, surveillance, eradication, and containment**							
Scenario 1*	174	43	35	9	194	48	404
Scenario 2**	157	43	30	8	175	48	363
Scenario 3***	133	44	32	11	135	45	300
**ICDM—Integrated crop and disease management**							
*Expert est. adoption scenario*	6	2	21	6	317	92	344
*50% reduced adoption scenario*	3	2	8	5	158	93	170
**CBRC—Conventional breeding of FWB resistant banana cultivars**							
*Expert est. adoption scenario*	201	34	18	3	373	63	593
*50% reduced adoption scenario*	101	34	9	3	187	63	297
**GMRC—Genetically modified FWB resistant banana cultivars**							
*Expert est. adoption scenario*	18	14	3	2	106	83	127
*50% reduced adoption scenario*	9	14	2	2	53	83	63

The adoption area of ESEC represents the area after 25 years where losses could be avoided due to the execution of the quarantine and surveillance measures at a national level *Doubled arrival time and 50% reduced increase of spread rate (12.50%) once Foc reaches the country as compared to a scenario without intervention; **Arrival time as in Scenario 1 minus 5 years; 50% reduced increase of spread rate (12.50%) once Foc reaches the country; ***Arrival time as in Scenario 1 minus 5 years; 25% reduced increase of spread rate (18.75%) once Foc reaches the country.

### Sensitivity Analysis

In addition to the adoption scenarios included in results (Tables 5, 6), we conducted sensitivity analysis for the pace of adoption and size of the effects. We also computed impacts for an even more conservative adoption scenario (25% of expert estimated adoption). The results of these additional scenarios are displayed in Table 5 for ESEC and Table 7 for the other options.

**TABLE 7:** Sensitivity analysis—benefits under different adoption scenarios.

**Technology**	**Benefits of lower adoption (25% of estimate)**		**All benefits (based on50% lower adoption scenario)**							
			**Scenario I: *Adoption delay of 2 years***		**Scenario IIa: *25% reduced effect***		**Scenario IIb: *50% reduced effect***		**Scenario III: (I+IIb) *Adoption delay + 50% reduced effect***	
	**NPV (US$’000)**	**IRR (%)**	**NPV (US$’000)**	**IRR (%)**	**NPV (US$’000)**	**IRR (%)**	**NPV (US$’000)**	**IRR (%)**	**NPV (US$’000)**	**IRR (%)**
ICDM	230,709	24	329,066	26	332,224	27	160,871	22	97,208	18
CBRV	66,937	15	19,155	12	124,657	18	66,148	15	-15,103	8
GMRV	63,055	23	80,352	24	99,872	26	62,812	23	34,606	19
***Note:*** *NPV calculated using a real interest rate of 10%.*										

Even under these increasingly, extremely, and conservative scenarios, all but one assessed research options yield positive NPVs and IRRs well above the 10% benchmark level (Tables 5, 7). These findings confirm our conclusion that investments in the assessed FWB research options generate positive economic and poverty reduction effects.

## Discussion and Conclusion

All four assessed research lines yield positive IRRs, ranging from 11 to 36%, suggesting that investment in research to reduce the impact of Foc TR4 is worthwhile. Even under scenarios of delayed uptake or lower adoption ceiling, benefits were robustly above the 10% benchmark. The ESEC and CBRC lines have the largest potential for poverty reduction since they cover more cultivars and more countries, particularly in Africa.

While these positive results are useful evidence for donors and development institutes and national research programs to include research on Foc TR4 among their priorities, the approximate nature of the research costs and the uptake costs mean that the results cannot be used for fine grain decision making about research priorities. Decision makers may be reluctant to base their decisions to fund one research approach rather than the other three based on these calculations. To make the results more useful for decision-making about research investments, the analysis could be improved by converting from global or aggregate country level to more targeted, location or context specific assessments.

The usefulness of disaggregation to refine results was demonstrated in our division of banana production by cultivar groups. This allowed us to differentiate rate of loss to Foc TR4 and to target different countries based on our prior knowledge of the production systems thereby differentiating uptake among the different research lines by both of these variables. In our initial research design, we planned to employ spatial data on banana production at the sub-country scale, but such data were not available at the time of the study. To improve granularity, the priority assessment cost-benefit analysis requires geographic distribution of banana production within country by cultivar group, production system, market destination, and potentially even degree of organization among growers. This last dimension has been highlighted by Montiflor et al. (2019) in their analysis of the institutional dimension of management of response to the outbreak and spread of Foc TR4 in the Philippines.

The availability of more detailed spatial data of banana production by cultivar, production system, and market is also a necessary resource to improve the projection of losses beyond our simple risk index model. The degree of dispersion of banana production areas and their location in river plains or on uplands have large potential effects on the rate of disease spread. Other production system characteristics like the quality of seed, the use of inputs which accelerate Foc inoculum build-up such as ammonium-based fertilizers and herbicides (Dita et al., 2018), and the nature of post-harvest packing and transport can also be incorporated into spatial models to generate more realistic scenarios of losses. Such a framework could also generate insights into the risks and mechanisms for dispersion of Foc TR4 from Cavendish monocrop production systems where most of the losses have occurred to smallholder diversified production areas whether contiguous or distant. Field studies are needed to build scenarios through modeling to indicate whether Foc TR4 is a Cavendish problem or can be projected to cause progressive losses in more diversified smallholder banana production as we have suggested in the loss model.

The information base to not only improve loss projections, but also segment growers of bananas and plantains based on more detailed characterization should facilitate the contrast among investments in the research lines by user groups. Research costs can be estimated with greater specificity than our general costs based on CG center working budgets. This segmentation would also provide the opportunity to make more detailed calculations of the dissemination costs. In our calculations, we have used a single estimate for all countries (following the standard value used across the different root and tuber research investments included in the wider RTB priority assessment study). In addition to the one-time dissemination costs, we have proposed annually recurring maintenance costs to surveillance operations. However, the investments required to upgrade and maintain national plant protection operations are certain to vary from country to country. Countries like Costa Rica and Mexico already have strong tradition and capacity, while other countries are much more incipient. Farm scale biosecurity measures (Kukulies and Veivers, 2017) are an additional dimension of costs to be included, although very little documentation exists of costs. A large transnational company recently reported their initial investment in biosecurity measures to prevent Foc TR4 at US$800 per hectare with additional recurring costs to maintain foot baths and vehicle wash-down facilities in operation. At the same time, supermarkets concerned about future banana supplies have also been incorporating farm level biosecurity for FWB into their certification programs^8^. However, addressing farm scale biosecurity among small scale growers for national markets in diversified farms remains to be developed which will provide a better basis for costing.

Increased production costs were added in the case of ICDM for purchased inputs. These costs may vary depending on the degree and nature of resistance to FWB of the cultivar. The GMRC presents a different challenge to countries open to this technology. We have included Peru and Mexico in target countries, but in practice, they have generally strong regulation against any type of GM crops. An additional complexity is the possible differentiation between genetic modification and gene editing. Procedures and documentation on biosafety for cultivar registration are potential costs which may be needed for more targeted priority assessment calculations (see example in Ainembabazi et al., 2015).

To move beyond the contrast of investments in different research options, optimization analysis could be considered. The spatial models linked with economic analysis could be used to explore mixed investments of ESEC, ICDM, and the breeding options as the disease spreads. Such models may generate different strategies for Africa than for Latin America, both continents at early stages in Foc TR4 spread, but with quite different cultivar preferences and grower groups. Such an optimization exercise for Asia with already advanced spread may need a greater emphasis on the use of already infested soils than on ESEC, although internal exclusion remains important.

In conclusion, the priority assessment exercise provides evidence that investments in all assessed research lines to address the threat and projected losses from Foc TR4 will provide positive returns and contribute to poverty reduction. A more fine-grained estimate of costs and benefits to assess alternative research lines would require more complete characterization and spatial distribution of banana production systems, improved projection of losses, more site-specific costs of research, and more detailed calculations of costs of uptake and impacts on production costs and viability. Such exercises, applied in different regions and countries from Asia to Latin America, should serve not only to improve research efficiency, but also provide science-based documentation for the debates about the severity of the Foc TR4 threat and alternative ways to address it.

## Data Availability Statement

The datasets generated for this study are available on request to the corresponding author.

## Author Contributions

MD, CS, and LP identified and defined the Fusarium research options. DP and LS conducted the economic analysis and all other data tasks. CS and DP did the final manuscript writing based on the existing RTB working manuscript. All authors contributed to the article and approved the submitted version.

## Conflict of Interest

The authors declare that the research was conducted in the absence of any commercial or financial relationships that could be construed as a potential conflict of interest.

## Footnotes

1 e.g., D. Koeppel (2011) in The Scientist: The beginning of the end for Bananas; Popular Science (2014): Has the end of the banana arrived?; P. Tullis in The Washington Post (2017): Bananapocalypse; F. Kentish (2015) in The Telegraph: Is this the end of the banana?; N. Fleming (2018) in The Guardian: Science’s search for a super banana (see reference list for full citations).

2 www.cgiar.org

3 http://www.promusa.org/Tropical+race+4+-+TR4

4 FAOSTAT http://faostat.fao.org/

5 World Bank, http://data.worldbank.org/indicator

6 In line with previous studies on (potential) plant/animal disease outbreaks applying a cost-benefit analysis framework (e.g., Cembali et al., 2003; Wittwer et al., 2005; Breukers et al., 2008; Cook et al., 2013, 2015) the benefits of delaying or preventing FWB occurrence/spread in our assessment are the avoided losses/costs that would have occurred without the (research) intervention within the same timeframe.

7 In the time span between conducting our analysis and writing this paper, Foc TR4 has been detected in Colombia, with subsequent efforts and investments to confine and manage the disease. Once present in the LAC region, it is very possible that the disease will further spread and this threat will likely result in increased adoption of Foc-related technologies and management approaches since many of the key banana exporters are located in this region and will have a keen interest to protect/recover their production.

8 See, e.g., https://www.globalgap.org/uk_en/media-events/news/articles/Tesco-Takes-Crucial-Step-to-Protect-Global-Banana-Production/
